# The utility of micro-computed tomography for the non-destructive study of eye microstructure in snails

**DOI:** 10.1038/s41598-019-51909-z

**Published:** 2019-10-28

**Authors:** Lauren Sumner-Rooney, Nathan J. Kenny, Farah Ahmed, Suzanne T. Williams

**Affiliations:** 1 Oxford University Museum of Natural History, Parks Road, Oxford, UK; 2 Natural History Museum, Cromwell Road, London, UK; 3 Exponent International Ltd, London, UK

**Keywords:** X-ray tomography, Evolution, Animal physiology

## Abstract

Molluscan eyes exhibit an enormous range of morphological variation, ranging from tiny pigment-cup eyes in limpets, compound eyes in ark clams and pinhole eyes in *Nautilus*, through to concave mirror eyes in scallops and the large camera-type eyes of the more derived cephalopods. Here we assess the potential of non-destructive micro-computed tomography (µ-CT) for investigating the anatomy of molluscan eyes in three species of the family Solariellidae, a group of small, deep-sea gastropods. We compare our results directly with those from traditional histological methods applied to the same specimens, and show not only that eye microstructure can be visualised in sufficient detail for meaningful comparison even in very small animals, but also that μ-CT can provide additional insight into gross neuroanatomy without damaging rare and precious specimens. Data from μ-CT scans also show that neurological innervation of eyes is reduced in dark-adapted snails when compared with the innervation of cephalic tentacles, which are involved in mechanoreception and possibly chemoreception. Molecular tests also show that the use of µ-CT and phosphotungstic acid stain do not prevent successful downstream DNA extraction, PCR amplification or sequencing. The use of µ-CT methods is therefore highly recommended for the investigation of difficult-to-collect or unique specimens.

## Introduction

Micro-computed tomography (µ-CT) is one of the most important developments in morphological research of recent decades. Non-invasive imaging facilitates the study of internal anatomy without causing disruption, distortion or damage to surrounding tissues, and methods including computed X-ray tomography (CT, μ-CT, nano-CT and synchrotron tomography) and magnetic resonance imaging (MRI) have enabled the dawn of ‘big data’ morphology. High-powered tomographic methods can provide resolution comparable to traditional histological techniques e.g.^1,2^ at much greater speed, with scans generally taking only a few hours to complete. Staining protocols may take several days, but are not labour-intensive. Conversely, histological sectioning can require several days of tissue preparation, staining and embedding prior to sectioning^3^.

An area of particular impact for penetrative imaging is the use of precious specimens in morphological studies^4,5^. Rare species, material collected from inaccessible localities, type or reference specimens, and historic museum collections are often limited in their availability for conventional, destructive techniques such as histology and dissection. Non-invasive scanning methods offer the opportunity to study such material with little or no risk to its integrity, enabling researchers to draw data from more specimens and larger regions of interest. However, there are still some barriers to the universal use of tomography in these cases. Different stains can be considered destructive to some extent; for instance phosphotungstic acid (PTA) is not broadly thought to be reversible, and even iodine-based staining cannot be removed completely (although some studies have demonstrated potential for removing both stains)^6^. This can cause scanning procedures to be classified as destructive, and therefore its use has been discouraged for important specimens. Additionally, the effects of both staining and scanning protocols on the viability of subsequent genetic analyses remain unclear^5,7–9^, though it appears that µ-CT itself does not compromise the viability of DNA or negatively affect PCR amplification^5,10,11^. In the age of genetics, this is an extremely important consideration, and while X-ray-related DNA damage has been studied in a handful of phyla, it remains a concern and can affect specimen availability. Finally, natural history collections frequently use 70–99% ethanol to preserve live-collected samples. The use of alcohol as a preservative can cause dehydration and distortion of the tissue, particularly over long periods, though Sombke *et al*.^12^ reported only slight effects on the appearance of neural tissue in a comparative study.

The improving speed and resolution of µ-CT scanning enables increasingly focussed morphological studies targeting specific organs and tissues, and in smaller subjects^13–17^. Initial studies on molluscs have also proven to be useful for examination of anatomical details in bivalves^18^. Of particular interest to this study is the soft-tissue visualisation and imaging in invertebrates, including central nervous and sensory systems^2,12,19–23^. Using synchrotron tomography, Taylor *et al*.^2,23^ and Wilby *et al*.^22^ reconstructed bee ocelli and compound eyes at sufficient resolution to analyse their visual properties, but to date we are unaware of any studies using µ-CT to examine evolutionary changes to visual systems across multiple taxa. Subtle changes to eye structure and innervation may only be apparent at fine resolution, previously achieved using classical histological techniques but now available through X-ray tomography. These fine details are crucial to comparative studies and the reconstruction of ancestral states in visual systems.

One area of longstanding interest in the field of visual evolution is that of eye loss. Lineages inhabiting dark environments such as caves, the deep ocean, and subterranean burrows often demonstrate apparently convergent eye loss, but the evolutionary mechanisms underlying this seemingly dramatic change are still the subject of significant debate^24,25^. Comparative morphological studies of eyed and eyeless lineages enable the reconstruction of ancestral eye characters and evolutionary trajectories in independent instances of eye loss; they are a valuable tool deployed alongside transcriptomic and developmental comparisons^26–28^. However, the taxa of interest for such research are often by nature difficult to obtain, especially in cavernicolous and bathybenthic groups. In many cases, taxa are described from a handful of specimens and as such are often unsuitable for histological study. The study of eye loss therefore stands to gain much from the application of non-destructive tomographic imaging, as resolution, speed and accessibility improve.

The gastropod family Solariellidae is a case in point, being a radiation of small, poorly-known, deep-sea snails that, while not uncommon at some localities, are sparsely represented in most collections^29,30^. They are of particular interest to research on the evolution of eye loss because they demonstrate multiple independent losses of vision in several deep-sea lineages^26,29^. Three solariellid gastropod species were selected for this study to compare morphological and genetic data obtained from specimens before and after PTA staining and µ-CT scanning. The specimens used in this study were previously also used for histological serial sectioning and tomographic reconstruction of the left eye^26^, providing an excellent opportunity for direct within-individual morphological comparison. The specimens were originally fixed in ethanol, which is common procedure for museum biological collections and widely used for convenience in the field. A previous study^26^ identified fine-scale morphological indicators of eye loss to assess macroevolutionary patterns at the family level; here we tested whether comparable resolution could be obtained from scanned specimens as from histological sections in order to meet the same goals in future research.

## Results and Discussion

### Stains and scanning

**Figure 1:**
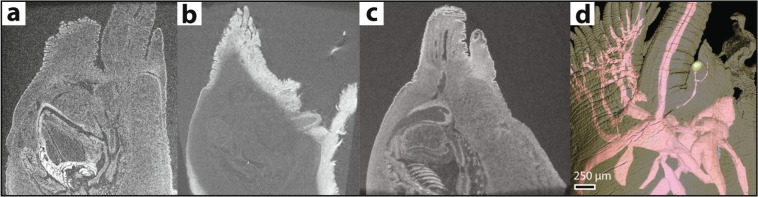
PTA staining substantially improves soft-tissue contrast in *Elaphriella wareni*. (**a**) Unstained biological tissues often have near-uniform X-ray absorption, giving low contrast in µ-CT scans and inhibiting their distinction. Inset: *Ilanga navakaensis*. Detail of eye from unstained specimen. (**b**) Contrast-enhancing stains such as phosphotungstic acid (PTA) have differential affinity to tissue types and facilitate their distinction, but incomplete penetration (as shown here) resulting from insufficient staining times may further inhibit feature reconstruction due to artefactual contrast variation across the sample and consequent problems in digital contrast enhancement. (**c**) Complete penetration by stain (13–14 days in this study) enables the distinction of additional tissues and at finer scales. (**d**) Reconstruction of fine neural structures based on PTA stained sample in (**c**).

Specimens used to evaluate eye morphology were collected in 2005–2006 and preserved in 96–99% ethanol. In larger specimens stained for less than 14 days, PTA penetration was incomplete, and many internal features were difficult to identify (Fig. 1). The resulting contrast variation across the sample meant structures were more difficult to reconstruct than in completely unstained specimens (Fig. 1). The results suggest that ethanol-preserved specimens 3–5 mm^3^ should be left for a minimum of 14 days in PTA prior to scanning. One particular benefit of using PTA stain is that longer exposure does not result in overstaining as with some other staining media, e.g. iodine^31,32^.

**Figure 2:**
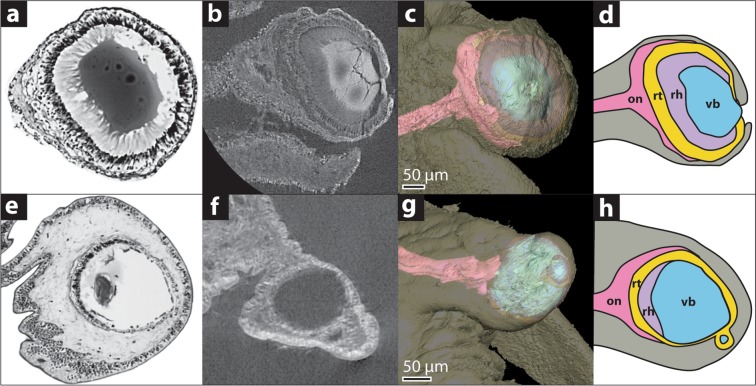
Micro-CT scans can produce comparable results to conventional histological techniques. (**a**–**d**) *Ilanga navakaensis*, an eyed shallow-water species. (**e**–**h**) *Bathymophila diadema* a deep-water species exhibiting eye reduction. (**a**,**e**) Histological sections. (**b**,**f**) μ-CT slices after PTA-staining. (**c**,**g**) Tomographic reconstructions based on μ-CT. (**d**,**h**) Schematic diagrams based on histological sections.

As expected, material that was sufficiently stained with PTA showed much better contrast than unstained material; different tissue types were easily distinguished in all fully stained specimens, including epithelial layers, neuropil and neuronal somata, and the individual components of the eye. In high-resolution scans (voxel size 0.7–0.8 µm), the projection of retinal microvilli is recognisable as striations in the eye cup (e.g. *Ilanga navakaensis* (=*Ilanga* 6 in^26,29^), Fig. 2b). However, a great deal more detail than expected was visible in scans of unstained specimens, due to enhanced phase-contrast during imaging, with nerve tissue and several different tissues of the eye visible (Fig. 1a). Owing to poor contrast and reduced ability to identify tissue types, the use of unstained specimens is most appropriate in cases where the fundamental structure of the region of interest is already known from the use of staining or histological sectioning on related species or representative samples. For example, while we were able to identify most major structures in the current samples, these have been studied at the histological level in the past, providing significant context for the interpretation of the low-contrast scans.

### Eye structure

Three specimens were used for μ-CT imaging and sequencing. *Ilanga navakaensis* lives in shallower water (collected live from 79–150 m^33^) and has intact eyes, with typical trochoid structure; *Elaphriella wareni* and *Bathymophila diadema* inhabit deep water (collected live from 782–957 m and 367–957 m respectively^29,34^) and both exhibit eye degeneration to different degrees. Dehydration from storage in ethanol is a common limitation of studying museum material^12^, and the use of PTA can cause neural shrinkage (though this is less pronounced than for other commonly used stains^35^). In order to optimise comparisons between them, the specimens selected for this study are of similar age and have been treated identically since collection, but this potential limitation should be noted. Reconstructions of eye microstructure were largely in accordance with previous descriptions of rehydrated specimens^26^. Where observations differ, this has been indicated.

**Figure 3:**
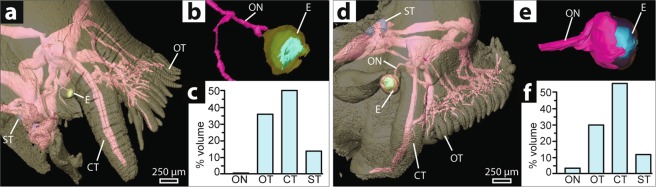
Sensory investment in *Elaphriella wareni* compared with *Ilanga navakaensis*. (**a**–**c**) *E. wareni* a reduced-eyed species. (**d**–**f**) *I. navakaensis* which has a typical vetigastropod eye. (**a**,**d**) Whole-head reconstructions. (**b**,**e**) Detailed reconstruction of eye. (**c**,**f**) Graph showing percentage of relative investment in sensory organs by volume for the optic nerve, oral tentacles and papillae (combined), cephalic tentacle and the volume of statocysts as a proxy for investment in this structure. Abbreviations: ON, optic nerve; OT, oral tentacles; CT, cephalic tentacle; ST, statocyst; E, eye.

*Ilanga navakaensis* has open pigment cup eyes typical of Vetigastropoda (Fig. 2). The eyestalk originates at the base of the cephalic tentacle and projects laterally and slightly antero-ventrally, terminating at 350 µm in length (Fig. 3). The eye is located at the tip of the eyestalk and eye diameter is measured at 270 µm, taken at the widest point of the retinal volume along an axis perpendicular to the long axis of the eyestalk, with a maximum aperture diameter of 40 µm. The retina forms a cup around 150 µm deep and 270 µm across, is composed of columnar cells and is darkly pigmented (pigment visible superficially and in histological sections). Projecting from the retina, a striated microvillous layer surrounds the vitreous body, which is thought to function as a lens^36^. Nerve tissue surrounds the proximal side of the retinal cup, which appears to be dually innervated by one larger (diameter 40 µm) and one smaller (diameter 20 µm) nerve; in previous reconstructions only one nerve was observed^26^. The larger nerve connects to the cerebral ganglion; the smaller nerve also projects towards the cerebral ganglion but could not be traced to a connection (Fig. 3).

*Elaphriella wareni* exhibits substantial degradation of the typical vetigastropod eye structure. Retinal pigmentation is absent on external examination, and the eye aperture is covered over by epithelium, sealing the retinal cup. The vitreous body appears to be fragmented into several distinct pieces interspersed with microvilli, rather than forming a single structure at the lumen of the eye. This is in contrast with Sumner-Rooney *et al*.^26^ where the vitreous body was thought to be entirely absent. The eye measures 120 µm in diameter at its widest point and 140 µm deep, with the apex of the eye lying 40 µm beneath the surface of the eyestalk. The optic nerve is narrow (diameter 20 µm) and was not seen to surround the proximal side of the retinal cup, in contrast to previous findings^26^ but remains connected to it at the basal-most point. The optic nerve projects towards the cerebral ganglia but could not be traced to a connection, in conflict with Sumner-Rooney *et al*.^26^, but as this specimen was imaged at low resolution (voxel size 3–4 µm) for examination of the central nervous system (see discussion below), it is possible that these fine details were beyond the limits of resolution rather than truly absent. Secondary innervation of the eye was not observed.

*Bathymophila diadema* also shows signs of degeneration of the eye. While retinal pigmentation persists (visible superficially and in histological sections), the eye aperture has been engulfed by the surrounding epithelium, as in *E. wareni*, and the vitreous body is fragmented and irregular in shape as previously reported^26^. The microvillous layer of the eye is proportionally much thinner than in *I. navakaensis*, but the general organisation of the retina appears similar. The occlusion of the aperture also appears to have caused the isolation of a retinal ‘bud’, a small region that has pinched off from the main part of the eye, a feature not noted in Sumner-Rooney *et al*.^26^. The eye is measured at 110 µm in diameter and has a total depth of 120 µm (including the retinal bud). Optic nerve diameter is 35 µm, but the nerve was not seen to surround the retina as observed in *I. navakaensis*. A second, smaller nerve (diameter 15 µm) also appears to innervate the retina at a point ventral and proximal to the larger nerve. Although this nerve was observed in histological sections it was not traceable at the resolutions provided by light microscopy^26^.

### Nervous system

The non-destructive nature of μ-CT allowed us to gather data about the head and central nervous systems (CNS) of specimens that was impossible to observe using histological reconstructions without destroying the entire head of the specimen. We therefore imaged *Ilanga navakaensis* and *Elaphriella wareni* at a lower resolution (voxel size 3–4 µm) to examine the CNS and other aspects of the head in both a sighted species and a species with degenerate eyes. Reduction in the visual system can cause dramatic parallel changes in the CNS, most notably the degeneration of neural tissues dedicated to visual processing. The modelled CNSs are consistent with the common ground plan for gastropods and vetigastropods in both species. The cerebral ganglia are triangular, with the longest side oriented dorso-posteriorly. They are connected to each other by an anterior cerebral commissure dorsal to the oesophagus, and to the pedal ganglia by two connectives that surround the oesophagus. The pedal ganglia themselves are elliptical and elongated along the anterior-posterior axis, which is typical in vetigastropods and sometimes leads to their description as medullary cords^37^. The pleural ganglia are small and slender, closely associated with the pedal ganglia and connected to the cerebral ganglia by a long connective. The statocysts sit between the anterior ends of the pedal ganglia. Width of the head, taken at its narrowest point anterior of the cephalic tentacles, was measured as a proxy for total size, being 1.45 mm in *I. navakaensis* and 1.71 mm in *E. wareni*.

In other dark-living taxa, reduction of the visual system is often compensated for by increased investment in gustatory, olfactory or tactile sensory modes. Tentacles in molluscs have a wide range of sensory functions, and it is expected that deep-water solariellids rely more heavily on these as their eyesight degenerates. The cephalic tentacle, directly anterior to the eyestalk, is dually innervated by a large (diameter at half-length 90 µm in *I. navakaensis*, 110 µm in *E. wareni*) and a smaller (diameter at half-length 60 µm in *I. navakaensis*, 100 µm in *E. wareni*) nerve, both originating in the lateralmost region of the cerebral ganglion. This stands in contrast to studies of other vetigastropod cephalic tentacles where only a single nerve has been identified^38^, though it appears the two nerves observed here share a joint origin in the cerebral ganglion.

The oral surface of the solariellid snout has a papillate surface, with a characteristic fringe of tentacles around the margin of the oral disk^39,40^. The oral papillae are densely packed, sometimes exhibit pale pigmentation, and can number in the hundreds, whereas the oral tentacles are significantly longer and are present in lower numbers. These tentacles are used to sweep through the sediment for food and are capable of particle recognition and selection by grasping^39^. The fringing oral tentacles and oral papillae are individually innervated by branching network of nerves from one major nerve (100 µm diameter in *I. navakaensis*, 120 µm in *E. wareni*), originating in the anterolateral edge of the cerebral ganglia, and multiple smaller nerves (c. 40 µm in *I. navakaensis*, 50 µm in *E. wareni*) projecting from the anteroventral margin of the cerebral ganglia. It appears that many, if not all, of the oral tentacles and papillae are dually innervated, once by the major tentacle nerve and once by one of the smaller tentacle nerves (Fig. 3). This is clearest in the longer marginal tentacles (the largest and lateralmost) that form a fringe around the mouth, but can also be seen in some oral papillae. *Ilanga navakaensis* has an estimated 312 oral papillae, of mean diameter at half-length 62 µm (±1.09 µm standard error, n = 49). The outer (anterior) fringe of oral tentacles had a mean length of 37.2 µm (±1.9 µm standard error, n = 23). *Elaphriella wareni* has an estimated 172 oral papillae, of mean diameter 90.8 µm (±2.9 µm standard error, n = 33). The outer (anterior) fringe of oral tentacles had a mean length of 55.6 µm (±3.2 µm standard error, n = 11).

Reconstructed nerve volumes for the right-hand side of the head were calculated to give an estimate of sensory investment in the oral tentacles and papillae; in *I. navakaensis* this volume was 0.0114 mm^3^ (30% of total measured sensory investment), while in *E. wareni* it was 0.0225 mm^3^ (36%). In contrast, the volume of the optic nerve in *I. navakaensi*s was 0.0012 mm^3^ (3.1%) but just 0.00024 mm^3^ (0.4%) in *E. wareni*. The cephalic tentacle nerves, which act as mechanoreceptors and likely have a chemosensory function^38^, show slightly smaller percentage investment in *E. wareni*: 0.021 mm^3^ in *I. navakaensis* (55.2%) and 0.031 mm^3^ in *E. wareni* (50%). The statocyst, which detects orientation and gravitational direction, was slightly larger in *E. wareni* (0.0088 mm^3^, 13.9%) than in *I. navakaensis* (0.00441 mm^3^, 11.6%) (here volume of statocyst was used as a proxy for investment in this structure). Even accounting for the slightly larger size of *E. wareni* than *I. navakaensis* (by a factor of 1.18), it appears that there is greater investment in the size and innervation of oral tentacles and papillae and in the size of the statocyst in *E. wareni*, but perhaps lesser investment in the cephalic tentacles. Although there are fewer oral tentacles and papillae in *E. wareni*, they are proportionally longer and broader than those of *I. navakaensis*.

Although taken from only two specimens, quantitative comparisons of neural investment in different sensory structures support the expectation that reduced-eyed deep-sea species will compensate for the loss of vision by increasing sensitivity of other sensory modes (Fig. 3). These comparisons should be interpreted with some care, given the small sample size and that both specimens have been stored in ethanol for several years which may affect volume and that PTA has been reported to cause shrinkage to neural tissue^35^. In mitigation, both specimens were collected and preserved in exactly the same way, within <2 years of each other, and are of very similar size and are likely to be similar in tissue composition. One effect of dehydration can be seen in *I. navakaensis* (Fig. 2b), where the vitreous body exhibits some cracking, but soft tissue features such as the overall concentric structure of the retina, which can be easily disrupted by dehydration, are intact. Although we cannot account for the effect of storage in alcohol on volume of neural tissue, the application of PTA staining did not affect the relative volumes of the cerebral ganglia and the eye cup in *I. navakaensis*, which were the same in stained and unstained scans. Further, measurements of optic nerve and overall eye diameter taken from μ-CT scans are in line with those taken from rehydrated and non-PTA stained tissue from the other eye in the same specimens^26^.

### PTA staining, scanning and DNA integrity

**Figure 4:**
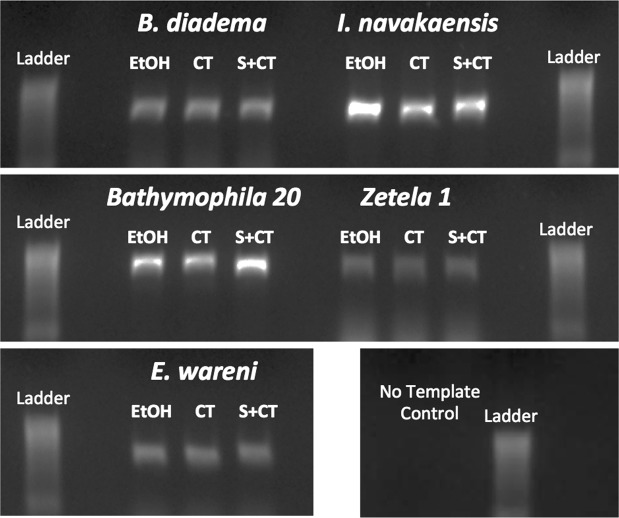
Staining and scanning procedures have no impact on DNA quality. Gel image from PCR, showing no difference in band size of PCR amplicons from DNA extracted from untreated and treatment samples. Tissue subsamples from the same specimen were taken before staining and scanning (‘EtOH’), after scanning but without staining (‘CT’) and after both staining and scanning (‘S + CT’). Raw metrics for DNA quantity and quality and best BLASTn hits of sequences are available in Supplementary Data 2. Gel images in this figure have been cropped from different parts of the same gel. An unedited version of the same gel is available in Supplementary Data 4.

The impact of PTA staining and µ-CT scanning on sample DNA is summarised in Fig. 4 and in Supplementary Data 2 and 3. It is clear from these results that neither PTA staining nor µ-CT scanning had any observable impact on DNA quality or quantity or PCR amplification. In only one case were the concentration and absorbance ratios markedly less impressive after µ-CT scanning and PTA stain than the baseline sample (*E. wareni*; Supplementary Data 2). Given that no other sample showed any impact from treatment, it is likely that this decrease in metrics is due to stochastic effects or a reduced amount of tissue used in this extraction rather than an intrinsic effect of PTA or the µ-CT scanning process. Indeed, in many of the treated samples, the results were more impressive than in the untreated samples.

Consistent results were obtained from PCR using both untreated and treated samples (Fig. 4). BLAST results of sequenced PCR products confirm the integrity of DNA post-scan, with the best BLAST hits in all cases to previously published sequences for the same species (Supplementary Data 3). No substantive DNA damage or PCR inhibition thus appears to have occurred as a result of either staining or scanning.

As previously seen with studies using alternative protocols and staining regimes^5,10^, it therefore seems that the ability to PCR amplicons is unaffected by standard µ-CT treatments. Here we show clearly for the first time that PTA also has no effect on DNA extraction or downstream reactions like amplification and sequencing, a finding that will ensure the applicability of this method to unique and precious samples, without any risk of loss of genetic information.

### Using µ-CT in the study of visual evolution and neuroanatomy

Our results confirm previous reports that μ-CT imaging is highly suitable for use on ethanol-preserved specimen collections, without adverse effects of X-ray exposure or PTA staining on DNA integrity, and with the potential to rival histological methods for the resolution of detail. The anatomical models reconstructed here accurately reflect those generated from histological slides, and in some cases facilitate the identification of new features such as the dual innervation of the retina. The potential manual errors in section production, collection and alignment are absent in X-ray tomography, and the ability to clearly re-slice image stacks along selected planes is a huge advantage, although there still exists the potential for related problems in μ-CT, including specimen movement and discrepancies between reconstruction methods. One clear advantage of the use of μ-CT is the possibility for examining a larger region of interest than using destructive methods; thus, we were able to incorporate the brain and oral and cephalic tentacles into this study, which were not included in Sumner-Rooney *et al*.^26^. These showed signs of slight differential neural investment in non-visual sensory modes between a sighted and degenerate-eyed species, with greater size and innervation of oral tentacles and papillae and larger statocysts in the latter. Given both the energetic savings of eye reduction and the loss of sensory information in this species, increased investment in chemo- or mechanoreceptors is in line with patterns observed in other eyeless taxa, most notably in troglomorphs e.g.^41^.

Conversely, some details were more difficult to identify using μ-CT; for example, although easily recognisable in histological slides, retinal pigmentation is not clearly identified in μ-CT scans, even in PTA-stained tissue. However, this feature can usually be viewed externally. In low-resolution scans, it was not possible to trace the projection of the optic nerve in *E. wareni*, which we know to be intact from histological study^26^. As there are no known ill effects from μ-CT imaging, specimens can be scanned multiple times at different resolutions to obtain both low- and high-resolution data (here, the entire head and a single eye). In *I. navakaensis* and *B. diadema*, which were scanned at high resolution, fine details of the eye were in line with histological descriptions^26^. Thus, we find that the quality of the scans is comparable to classical histological methods, but the availability of histological slides for these and related species is helpful for the identification of tissue types.

As μ-CT and nano-CT techniques continue to improve and become more economical, we anticipate that the study of fine structures such as eyes and ocular tissues will be widely available at resolutions comparable not only with histology and light microscopy but eventually with electron microscopy. Specimens from inaccessible localities or that are available in low numbers will undoubtedly benefit the most from this and future methodological developments, and the potential wealth of new data from such taxa is enormous. Recent advances in the field of computational optical analyses also open the door to functional modelling of eyes specifically. Taylor *et al*.^2,23^ and Wilby *et al*.^22^ have used synchrotron X-ray tomography to model insect eyes for ray-tracing analysis, modelling the path of light through the ocellar system and compound eyes to study their optical function. Such analyses could transform the way researchers study vision, especially in animals that are too rare or inaccessible for ethological experimentation, and that in many cases also require non-destructive examination.

## Conclusion

Here we demonstrate that non-destructive µ-CT methods provide adequate detail of eye microstructure when compared to histological techniques, and by the virtue of their nature can provide additional insight into wider neuroanatomical changes such as neural reinvestment in other sensory modes without incurring damage to rare or unique specimens. Furthermore, we confirm that samples that have undergone PTA staining and µ-CT can still be used for DNA extraction and gene amplification with no impact on the quality of result. The utility of this method for even the smallest of unique samples therefore seems clear: these samples can be preserved for future generations while still offering up insights to science today.

## Methodology

### Samples

Data for eye morphology are presented here for three solariellid specimens: *Ilanga navakaensis* (MNHN IM-2009-31831), *Bathymophila diadema* (MNHN IM-2007-18319), and *Elaphriella wareni* (MNHN IM-2009-18318), all from the Muséum National d’Histoire Naturelle (MNHN), Paris (for the context of collecting expeditions, see Bouchet *et al*.^42^ and http://expeditions.mnhn.fr). These same three specimens were previously used for traditional histological studies of eye structure^26^ (Supplementary Data 1). Note that newly published data suggest that *B. diadema* should be assigned to a new genus^43^.

### Micro-computed tomography

Preserved head and foot tissue samples were stained in 1% phosphotungstic acid (PTA) in ethanol for 12–14 days. Specimens were placed inside 200 µl PCR tubes and immobilised using plastic supports and 2% w/v ultrapure low melting point agarose (ThermoFisher) topped up with 70% ethanol. Three PCR tubes were placed one above the other inside a single 1000 µl pipette tip, and the regions of interest were imaged with a Zeiss Xradia Versa 520 (Pleasanton, United States) at the Natural History Museum, London. All specimens were scanned before and after staining with PTA. Low magnification scans (4x objective, voxel size 3–4 µm) to capture brain structure and tentacle innervation were conducted at 40 kV and 76 µA, taking 3201 projections with an exposure time of 5 s. The eyes of *I. navakaensis* and *B. diadema* were additionally scanned at higher magnification (20x objective, voxel size 0.7–0.8 µm) for direct comparison to histological sections taken previously^26^, at 60 kV and 84 µA, taking 3201 projections with an exposure time of 15 s. Enhanced imaging contrast from staining with PTA was further improved by using increased sample-detector distance during scanning allowing for the possibility of visualising phase contrast fringes. This combined force gave the ability to distinguish between many types of soft tissues. Reconstructed scans were processed and cropped in Fiji^44^ before segmentation and analysis in Amira (FEI, Thermo Fisher).

### DNA extraction and sequencing

Samples of tissue from the three samples described above, and two additional specimens from the same family, but representing different genera, which were scanned contemporaneously (*Zetela* 1, MNHN IM-2009-8748; *Bathymophila* 20, MNHN IM-2009-23102; µ-CT data not shown), were used to assess the impact of PTA staining and µ-CT imaging on DNA. Tissue subsamples from the same specimen were taken before staining and scanning (‘EtOH’ samples), after scanning but without staining (‘CT’) and after both staining and scanning (‘stain + CT’). All samples were a maximum of approximately 5 mm^3^ before DNA extraction, with some smaller samples present.

DNA was extracted using a DNeasy minikit (Qiagen) according to the manufacturer’s animal tissue protocol, with the following notes and exceptions. Separately supplied, fresh Proteinase K (20 mg/ml, Qiagen) was used for digestion. Tissue was ground manually for approximately 20 seconds per sample with plastic micropestles (VWR) prior to a 4-hour digest at 56 °C. Thereafter, the protocol was unchanged. DNA concentration and absorbance were measured on a Nanodrop ND-1000 (Thermo-Fisher).

For PCR, DNA concentration was normalised to approximately 26 ng/µL (the concentration of the second-most dilute sample), with the sole exception of the *E. wareni* ‘stain + CT’ sample, which was less concentrated. An aliquot of 1 µL for 14/15 samples was then used for PCR. For the *E. wareni* ‘stain + CT’ sample alone, 13 µL of DNA was used for PCR, resulting in a final volume of 26 ng of DNA per 50 µL reaction for all samples.

A PCR was run with universal primers for 16 S rRNA (16Sar and 16Sbr^45^), with an expected band size of approximately 600 bp. Thermal cycling conditions were: 94 °C for 3 mins, then 33 cycles of 94 °C for 30 sec, 50 °C for 30 sec, 72 °C for 45 sec, then a final extension at 72 °C for 5 min, followed by a 10 °C hold. PCRs were run alongside a 100 bp ladder (NEB) on a 1% w/v agarose gel using GelRed dye (Biotium). Samples were manually cut from the gel, and the ‘stain + CT’ sample for each specimen was gel extracted using a QiaQuick gel extraction kit (Qiagen). Samples were then sequenced using the Sanger method and the same primers as used for PCR, on a 3730xl DNA analyser (Applied Biosystems) at the Molecular Laboratory, the Natural History Museum, London. To confirm the sequence homology of our samples, we used BLASTn on the online NCBI BLAST server^46^.

## Supplementary information

Supplementary Information 1-4
