# Supplementary material for: The utility of micro-computed tomography for the non-destructive study of eye microstructure in snails
Source: Sci Rep. 2019 Oct 28;9:15411. doi: 10.1038/s41598-019-51909-z (PMC6817935; doi:10.1038/s41598-019-51909-z)
Supplement: Supplementary file 1 — Supplementary Information 1-4 [file 41598_2019_51909_MOESM1_ESM.pdf]

The utility of micro-computed tomography for the non-destructive study of eye microstructure in snails

Lauren Sumner-Rooney[1], Nathan J. Kenny[2], Farah Ahmed[2,3], Suzanne T. Williams[2]*

[1]*Oxford University Museum of Natural History, Parks Road, Oxford, UK*

[2]*Natural History Museum, Cromwell Road, London, UK*

[3]*Exponent International Ltd, London, UK*

*Correspondence: Suzanne T. Williams; email: s.williams@nhm.ac.uk

Supplementary Files:

Supplementary Data 1: Sampling details for specimens used in this study including locality data, latitude and longitude, depth, MNHN expedition, station number and registration number.

Supplementary Data 2: Raw metrics regarding DNA quantity and quality, both before and after standard PTA treatment for μ-CT scanning. Tissue subsamples from the same specimen were taken before staining and scanning ('EtOH'), after scanning but without staining ('CT') and after both staining and scanning ('S+CT').

Supplementary Data 3: Best BLASTn hits of resulting F/R sequences resulting from PCR of samples. Tissue subsamples from the same specimen were taken before staining and scanning ('EtOH'), after scanning but without staining ('CT') and after both staining and scanning ('S+CT').

Supplementary Data 4: Unedited gel image used to produce Figure 4.

Supplementary Data 1. Sampling details for specimens used in this study. Specimens marked with an asterisk were also used in histological studies (Sumner-Rooney et al. (2016). All samples have been included in molecular phylogenetic studies (Williams et al. 2013; Sumner-Rooney et al. 2016).

Species	Sampling locality	Registration Numbers
*Bathymophila diadema**	SE Fairway, New Caledonia; 21° 29' S, 162° 36' E; 883-957m; EBISCO, CP2651	MNHN IM-2007-18319
*Ilanga navakaensis **	E Aoré Island, off Aimbuei Bay, Vanuatu; 15°34.9'S, 167°13.9'E; 10-51m; SANTO2006, EP35	MNHN IM-2009-31831
Bathymophila 20	Ile Matthew-Volcan, New Caledonia; 22°19'S, 171°20'E; 925m; EXBODI, DW3879	MNHN IM-2009-23102
*Zetela 1**	Mozambique Channel; 25° 13' S, 35° 21' E; 700-707m; MAINBAZA, CP3138	MNHN IM-2009-8748
*Elaphriella wareni**	SE Fairway, New Caledonia; 21°29'S, 162°36'E; 883–957m; EBISCO, CP2651	MNHN IM-2009-18318

Supplementary Data 2. Raw metrics regarding DNA quantity and quality, both before and after standard PTA treatment for µ-CT scanning. Tissue subsamples from the same specimen were taken 1) before staining and scanning ('EtOH' samples), 2) after scanning but without staining ('CT'), and 3) after both staining and scanning ('stain+CT').

	Concentration, ng/uL, 4 s.f.			A260/280			A260/230		
Sample	EtOH	CT	stain + CT	EtOH	CT	Stain + CT	EtOH	CT	stain + CT
Bathymophila diadema	160.3	1390	55.49	1.93	2.02	1.93	1.93	1.95	1.88
Ilanga navakaensis	392.3	2297	96.26	1.95	1.89	2.02	2.3	1.73	2.1
Bathymophila 20	11.68	947.9	26.66	1.43	1.89	1.98	1.02	2.23	1.61
Zetela 1	567	1515	95.56	1.96	1.96	1.98	1.95	1.93	1.69
Elaphriella wareni	415.3	784.6	1.706	2.01	2.05	0.92	2.2	2.21	0.25

Supplementary Data 3: Best BLASTn hits of resulting F/R sequences resulting from PCR of samples. Tissue subsamples from the same specimen were taken before staining and scanning ('initial' samples), after scanning but without staining ('CT') and after both staining and scanning ('stain+CT').

Sample	F and R sequence hit	*E* value
Bathymophila diadema	*Bathymophila diadema* mitochondrial partial 16S rRNA gene, MNHN:200718320, isolate 10546_EBI	0
Ilanga navakaensis	*Ilanga* sp. 6 LS-2013 mitochondrial partial 16S rRNA gene, MNHN:200718446, isolate AT112_BC4953	0
Bathymophila 20	*Bathymophila* sp. 20 LS-2016 mitochondrial DNA 16S-23S, isolate 23102_JS190.EXB, MNHN_2009_23102	0
Zetela 1	*Zetela* sp. 1 LS-2013 mitochondrial partial 16S rRNA gene, MNHN:200915167, isolate 15167_254_MBZ	0
Elaphriella wareni	Solariellidae clade C sp. 2 LS-2013 mitochondrial partial 16S rRNA gene, MNHN:200718318, isolate 10509_EBI	0

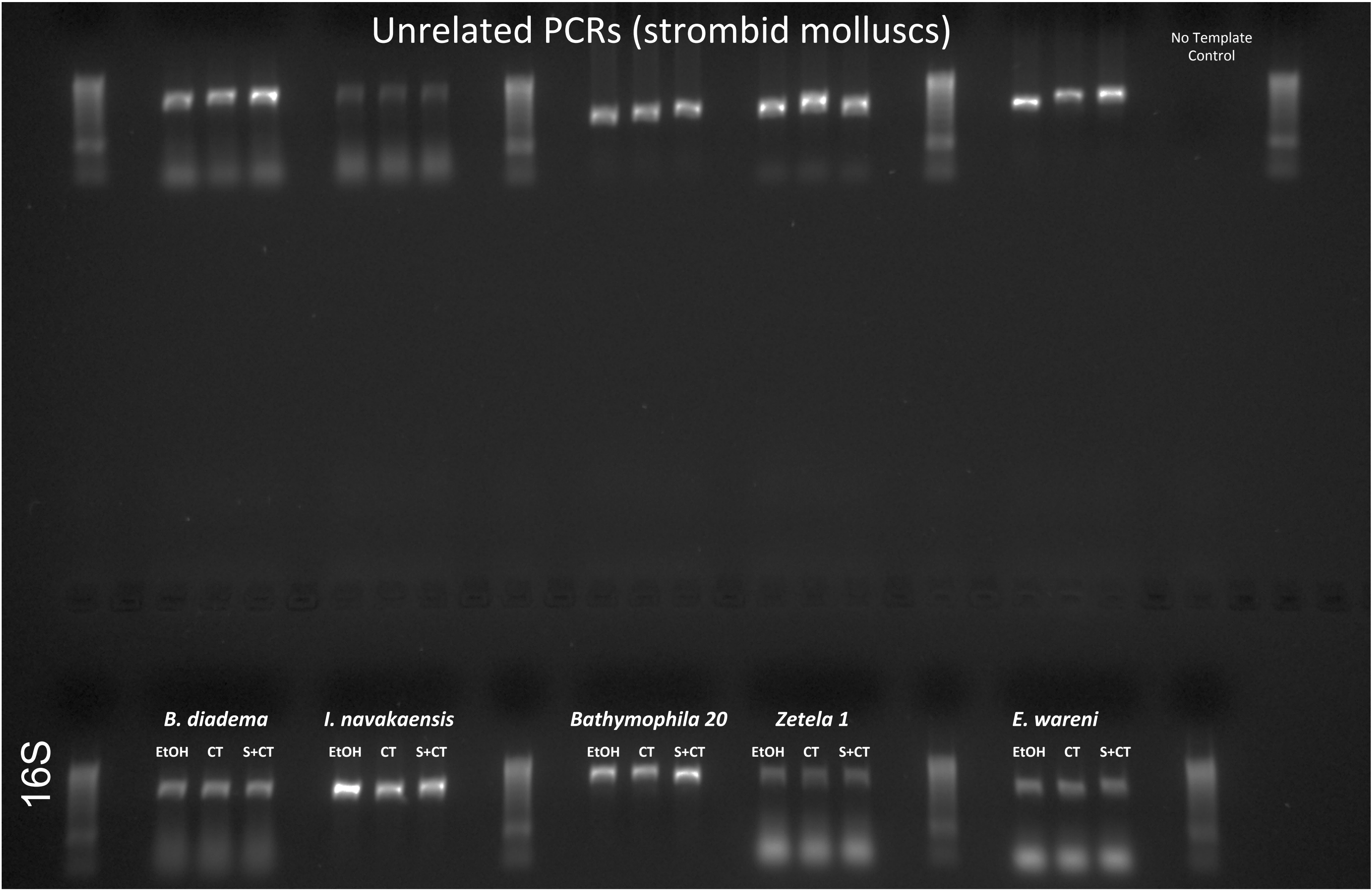
Unrelated PCRs (strombid molluscs)
No Template Control
16S
B. diadema
EtOH CT S+CT
I. navakaensis
EtOH CT S+CT
Bathymophila 20
EtOH CT S+CT
Zetela 1
EtOH CT S+CT
E. wareni
EtOH CT S+CT
